# In vitro attrition wear resistance of four types of paste-like bulk-fill composite resins

**DOI:** 10.1186/s12903-022-02393-x

**Published:** 2022-08-21

**Authors:** Faeze Asadian, Amirahmad Pahlavan Hoseini, Leila Ahmadian, Niyousha Rafeie, Samaneh Rezaei, Zohreh Moradi

**Affiliations:** 1 Restorative Dentistry Department, School of Dentistry, Birjand University of Medical Sciences, Birjand, Iran; 2 Prosthodontics Department, School of Dentistry, Islamic Azad University of Medical Sciences, Tehran, Iran; 3 Advanced Prosthodontics Program, College of Dentistry, University of Illinois at Chicago, Chicago, USA; 4 Dental Research Center, Dentistry Research Institute, School of Dentistry, Tehran University of Medical Sciences, Tehran, Iran; 5 Restorative Dentistry Department, School of Dentistry, Qom University of Medical Sciences, Qom, Iran; 6 Restorative Dentistry Department, School of Dentistry, Tehran University of Medical Sciences, North Amirabad Ave, Tehran, Iran

**Keywords:** Composite resins, Filtek bulk fill, Tooth wear, Tooth attrition, Filtek Z250

## Abstract

**Background:**

Recently, the application of bulk-fill composite resins has increased significantly. Attrition wear and the consequently increased surface roughness of composite resins are among the causes of restoration failure in the posterior teeth. This study aimed to compare the attrition wear and surface roughness of four types of bulk-fill composite resins compared to a conventional composite resin.

**Methods:**

EverX-Posterior, X-tra fil, SonicFill 2, and Filtek Bulk-Fill composites (bulk-fill) and Z250 composite (conventional resin composite) were evaluated. Thirty cylindrical specimens (n = 6) were weighed and monitored for 24 h until their weight was stabilized. The primary surface roughness of the specimens was measured by a profilometer. The specimens were then subjected to attrition wear in a chewing simulator. Next, the specimens were weighed, and the surface roughness was measured again. Data were analyzed by one-way ANOVA and Tukey’s post-hoc test at *P* < 0.05 significance level.

**Results:**

According to one-way ANOVA, the difference in weight loss was significant among the groups (*P* = 0.004) but the difference in surface roughness of the groups was not significant after the attrition wear (*P* > 0.05). Tukey’s post-hoc test showed that the weight loss of bulk-fill composites was not significantly different from that of Z250 conventional composite after the attrition wear (*P* > 0.05).

**Conclusion:**

Within the limitations of this study, it appears that the tested bulk-fill composite resins are comparable to the conventional composite regarding their attrition wear, increased surface roughness, and weight loss.

**Supplementary Information:**

The online version contains supplementary material available at 10.1186/s12903-022-02393-x.

## Background

The demand for esthetic tooth-colored dental restorations has greatly increased in recent years. Dental composite resins are highly popular among patients due to their tooth-like color and excellent esthetics. Also, they can bond to tooth structure, require conservative cavity preparation, and have low thermal conductivity. All these properties contribute to the increasing use of composite resins by dental clinicians [1]. In this regard, many attempts have been made to improve the physical and mechanical properties of composite resins [2, 3]. The introduction of bulk-fill composite resins was among the main advances made in the field of composite materials; these composite resins can be applied in thicker layers, due to their improved curing depth attributed to their large filler particles, decreased rate of pigments, and higher translucency. Therefore, these composites can be applied in 4-mm-thick increments and cured for 20 s with medium intensity to reach their optimal mechanical properties [4].

However, there are concerns regarding the application of composite resins in posterior areas due to their limited resistance to mechanical forces during clinical service in the oral environment. As a result, further knowledge about the wear processes in the oral environment can help improve the wear resistance of restorative materials [5].

The attrition wear is the physiological wear that occurs as the result of the contact of opposing teeth in absence of an abrasive agent. This type of wear increases by an increase in the amount of applied load and duration of load application. Also, the presence of a lubricant such as saliva affects the rate of wear [6]. Wear of composite resins may be related to several factors including the amount and size of filler particles, and chemical formulation of the resin matrix. Presence of the smaller size of the filler particles with a certain volumetric ratio decreases the space between the particles and results in lower wear. Moreover, the quality of the filler-matrix bond and proper curing of the resin matrix are among other influential factors in wear of composite resins [7–10]. In general, dental composites have filler particles larger than 1 µm, which have low resistance to attrition wear [11].

Several studies have compared wear properties of bulk-fill and conventional composites [12–14], however, the results of these studies are inconsistent. For instance, a previous study evaluating wear of four types of bulk-fill composite and one type of a conventional composite reported that wear resistance of bulk-fill composites was similar to that of a conventional composite [15]. On the other hand, Osiewicz et al. [16] reported a higher wear rate of bulk-fill resin composites compared to that of conventional ones.

Considering the novelty of bulk-fill composite resins and the lack of sufficient studies on this topic, as well as the significant role of wear resistance in long-term success of restorations and inconsistent results of the previous studies on this topic, this study aimed to compare the attrition wear and surface roughness of four types of bulk-fill composite resins in comparison with a conventional composite.

The first null hypothesis was that different types of resin composites are not significantly different regarding the surface roughness values. The second null hypothesis was that different types of resin composites are not significantly different regarding their attrition wear.

## Materials and methods

*This in-vitro*, experimental study was approved by the ethics committee of Tehran University of Medical Sciences (IR.TUMS.DENTISTRY.REC.1398.146). The minimum sample size required for each group was calculated to be 6 according to the results of Turssi et al., [10] assuming the effect size of 0.82, standard deviation of 7, α = 0.05, and β = 0.2 using the one-way ANOVA power analysis feature of PASS 11 software.

Four types of bulk-fill composite resins and one conventional composite have been used in the present study (Table 1). Six cylindrical specimens were fabricated from each type of composite resin for the wear test using plexiglass molds (an internal diameter of 10 mm, and thickness of 4 mm); bulk-fill composite resins were packed in the molds, a glass slab was placed over each mold and the specimens were cured using a polywave LED curing unit (Bluephase; Ivoclar Vivadent AG, Schaan, Liechtenstein) with 385–515 nm wavelength and 1200 mW/cm^2^ light intensity for 30 s. The light intensity was periodically checked by a radiometer (Kerr SDS, radiometer Optilux Model 100). For the control group, the conventional composite was placed in the mold of 2 mm thickness and irradiated from the top. The next increment was also applied with 2 mm thickness and cured.

**Table 1:** Characteristics of composite resins evaluated in this study

Brand name	Composite type	Manufacturing company	Composition	Filler percentage and size
Everx posterior	Short fiber composite	GC Corp., Tokyo, Japan	Short E-glass fiber filler, barium glass, Bis-GMA, PMMA, TEGDMA	74.2wt% 53.6vol% Hybrid filler fractions and E-glass fibers (1–2 mm length), Ba-B-Si glass filler (0.1–2.2 µm) [17]
Filtek BulkFill Posterior	Nano fill	3M ESPE, St. Paul, MN, USA	Non- agglomerated/non-aggregated 20 nm silica filler, non agglomerated/non-aggregated 4 to 11 nm zirconia filler, aggregated zirconia/silica cluster filler, ytterbium trifluoride filler consisting of agglomerate 100 nm particles, ERGP-DMA, diurethane-DMA, 1, 12-dodecane-DMA	76.5 wt% 58.4 vol% Fillers are a combination of zirconia and silica having a particle size of 0.01–4.5 microns and ytterbium trifluoride filler having a particle size of 0.1 -5.0 microns [18]
SonicFill 2	Nanohybrid	Kerr corp. Orange, CA, USA	Poly(oxy-1,2-ethanediyl), α,α′-[(1 methylethylidene)di-4, 1-phenylene]bis[ω-[(2- methyl-1-oxo-2-propen-1-yl)oxy]-Not available. 2,2′-ethylenedioxydiethyl dimethacrylate	81.3% wt % unreported Nanoscale zirconium oxide, silica oxide particles (10–30 nm) [19]
X-tra fil	Hybrid	VOCO Cuxhaven, Germany	Barium- boron- alumino- silicate glass, Bis-GMA, UDMA, TEGDMA	86 wt% 70.1 vol% 0.04–3 µm [20]
Filtek Z250 Universal	Microhybrid	3 M ESPE, St. Paul, MN, USA	Zirconia/silica without silane treatment, Bis-GMA, UDMA, Bis-EMA, TEGDMA	82 wt% 60 vol% 0.01 to 3.5 µm with an average particle size of 0.6 µm

Then, all specimens were removed from the molds and their upper surface was marked by placing a notch on each specimen. Then, the upper surface was polished with coarse, medium, and fine aluminum oxide discs (Sof-Lex; 3M ESPE, St. Paul, MN, USA). Each disc was used for 15 s. Next, the specimens were cleaned in an ultrasonic bath (containing distilled water) for 10 min and were then dried in an incubator at 37 °C. The specimens were weighed using a digital balance (Precision Scale, Solid, USA Lab Balance Analytical Digital) with 0.001 g accuracy every 24 h until their weight was stabilized. The primary surface roughness (Ra, Rq and Rz values) was also measured by a contact profilometer (TR-200; Time Group, USA) with 0.01 µm accuracy; for each specimen, the surface roughness was measured at 3 points with a 2.5 mm distance from each other. The mean of the three measurements was calculated as the surface roughness of the respective specimen.

The specimens were then subjected to 250,000 cycles (corresponding to one year of mastication in a normal individual) of attrition wear in a chewing simulator (C-S-4, SD-Mechatronik Company, Germany) [21]. For this purpose, the composite specimens were placed in Teflon molds corresponding to the size of the specimens (such that the specimens did not move during load application). Extracted sound human molars were also mounted in acrylic resin in Teflon molds and were positioned such that their occlusal surface opposed the composite specimens. For each new composite specimen, the natural tooth was replaced with a new one. The force load and range of motion were 50 N and 0.8 mm respectively [22]. In the chewing simulator, the specimens were immersed in artificial saliva with a composition of 1.5 mmol/L Ca^2+^, 50 mmol/L KCl, 0.9 mmol/L PO_4_^3−^, and 20 mmol/L trihydroxy methyl aminomethane, at a pH of 7 [23].

After the attrition wear, the specimens were rinsed with air and water spray for 1 min and placed in an ultrasonic bath for 10 min. Next, the specimens were dried in an incubator at 37 °C until their weight was stabilized. Finally, the specimens were weighed again and the surface roughness was measured for the second time. The weight loss of specimens indicated the rate of their attrition wear and was recorded.

Considering the normal distribution of data, data were analyzed using one-way ANOVA. Tukey’s post-hoc HSD test was used for pairwise comparisons. The level of significance was set at 0.05.

**Fig. 1:**
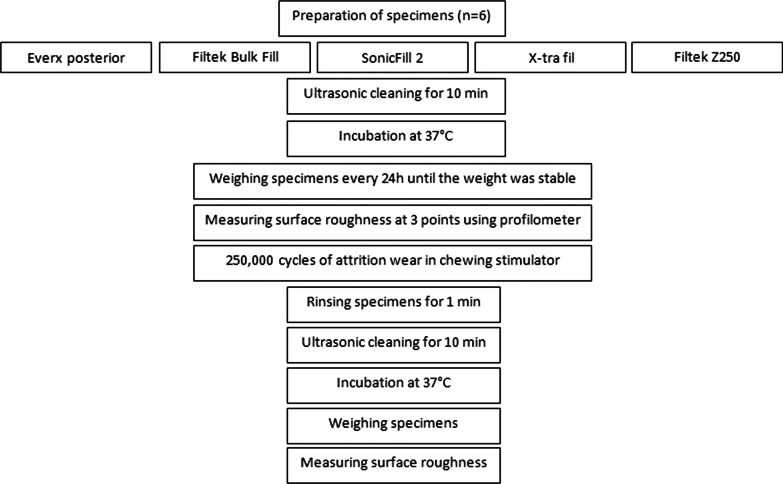
Methodology used in the present study

Figure 1 summarizes the methodology used in the present study.

## Results

Table 2 shows the descriptive values regarding the changes in weight and surface roughness of the specimens after the attrition wear. Filtek Bulk-Fill showed the highest Ra value followed by X-tra fil, EverX-Posterior, SonicFill 2, and Z250 respectively. The highest attrition wear was observed in X-tra fil composite, followed by Z250, SonicFill 2, EverX-Posterior, and Filtek Bulk-Fill respectively. One-way ANOVA revealed that the difference in weight loss was significant among the groups after the attrition wear (*P* = 0.004) while the difference in surface roughness was not significant (*P* > 0.05, Table 2).

**Table 2:** Measures of central dispersion regarding the changes in weight (mg) and surface roughness (µm) of specimens after the attrition wear (n = 6)

Type	Minimum	Maximum	Mean ± SD
Z250			
W. diff	− 8.00	− 2.00	− 4.3333 ± 2.422
Ra. diff	− 0.99	1.28	0.0480 ± 0.918
Rq. diff	− 1.09	1.57	0.1493 ± 1.146
Rz. diff	− 1.76	5.41	1.4027 ± 2.970
X-tra fil^a^			
W. diff	− 7.00	− 5.00	− 6.1667 ± 0.983
Ra. diff	− 0.17	1.85	0.6245 ± 0.726
Rq. diff	− 0.38	1.57	0.3465 ± 0.780
Rz. diff	− 0.51	4.88	2.1058 ± 1.763
EverX^b^			
W. diff	− 4.00	− 1.00	− 2.5000 ± 1.048
Ra. diff	0.18	1.08	0.5190 ± 0.329
Rq. diff	0.09	1.00	0.6010 ± 0.329
Rz. diff	− 0.04	4.45	1.9712 ± 1.686
Filtek Bulk-Fill^b^			
W.diff	− 3.50	− 1.40	− 2.3833 ± 0.798
Ra. diff	− 0.10	2.49	1.3623 ± 0.919
Rq. diff	0.19	2.22	1.3958 ± 0.753
Rz. diff	− 0.39	5.11	1.9093 ± 2.367
SonicFill 2			
W. diff	− 8.00	− 2.00	− 3.5000 ± 2.345
Ra. diff	− 1.14	2.00	0.3855 ± 1.310
Rq. diff	− 2.32	2.26	0.3223 ± 1.781
Rz. diff	− 5.42	6.50	0.6483 ± 4.200

Z250 and SonicFill 2 did not show statistically significance difference with other groups regarding weight loss. The same lowercase letter indicates lack of statistically significant difference between the two subgroups (*P* > 0.05). W: weight, Diff: difference

Regarding weight loss of specimens after the attrition wear, Tukey’s post-hoc test showed no significant difference between the Z250 conventional composite and other composite resins (*P* > 0.05). SonicFill 2, also, had no significant difference with any other composite either (*P* > 0.05). However, the difference was significant between X-tra fil and EverX-Posterior (*P* = 0.007) and X-tra fil and Filtek Bulk-Fill (*P* = 0.005); X-tra fil showed significantly greater weight loss compared to EverX-Posterior and Filtek Bulk-Fill after the attrition wear. EverX-Posterior and Filtek Bulk-Fill had no significant difference with each other (*P* > 0.05).

## Discussion

According to the results of the present study, no significant difference was observed between different types of composites regarding the surface roughness and thus, our first hypothesis was rejected. However, weight loss of specimens was significantly greater in X-tra fil composite compared to those of the other groups and our second hypothesis was accepted.

Roughness is described by many parameters including Ra, Rz, and Rq. Ra, Rz, and Rq are defined as the arithmetical average height, the difference in height between the average of five highest peaks and five deepest valleys, and the root mean square of the height values, respectively. It should be noted that measuring Ra is not sufficient for a comprehensive description of the surface and other parameters such as Rz and Rq should be evaluated as well [24]. Accordingly, the surface roughness of all composite types increased after the attrition test but this increase was not statistically significant. This finding was in contrast to the findings of Ting Ho et al. [25] who evaluated the surface roughness of composite specimens after chewing simulation. According to their results, the surface roughness parameters increased significantly after the test since wear exposed the fillers and subsequently increased the surface roughness. However, it should be noted that in their study, the change in surface roughness of resin composite after antagonist wear against monolithic zirconia and lithium disilicate ceramics was reported while in this study, a natural tooth was used as an antagonist in chewing simulator device. Due to the positive relationship between the hardness of ceramics and their abrasiveness, it is possible that zirconia and lithium disilicate increased the surface roughness of the composite since their hardness is greater than that of natural teeth used in the present study. Moreover, the size and distribution of filler particles in the resin matrix are among the important factors affecting the surface roughness of materials after wear [26, 27].

In the present study, the change in surface roughness of bulk-fill composites and a hybrid conventional composite was not significantly different. This result was in contrast to the findings of Oneil et al. [28] who reported that a bulk-fill composite had 2 to 7 times higher surface roughness than a conventional hybrid composite. This finding may be due to the fact that they used Admira Fusion x-tra as the bulk-fill composite. Hybrid composites have filler particles in the range of 40–300 nm in size. Due to the large size of filler particles and their irregular arrangement, they become exposed as the result of wear of the resin matrix and increase the surface roughness compared with conventional composite resins [13, 28].

Although the change in surface roughness of X-tra fil was not significant, it eventually showed a surface roughness above the clinically acceptable threshold, which can be due to the larger size of filler particles in this composite resin. Filler particles larger than 20 µm have also been incorporated into the composition of this composite resin since they enable better light penetration and enhance curing [29].

The current results revealed significantly higher weight loss due to attrition wear in X-tra fil bulk-fill composite compared with EverX-Posterior, and Filtek Bulk-Fill. This can be related to its higher surface roughness caused by the attrition test that probably resulted in the easier separation of superficial fillers exposed by the wear process and subsequently greater weight loss of this composite resin [13]. Greater wear of X-tra fil can also be due to its high filler percentage. Hu et al. [30] showed lower wear of composite specimens with a filler weight percentage less than 60%. The wear rate was higher in composite resins with 80%-87.5% filler weight percentage, which can be due to mass loss from the surface of specimens with high filler content. Mass loss occurs due to an increase in the friction coefficient between the filler particles and the matrix as well as the low bond strength between them. Mass loss can also result in higher surface roughness of these materials [30].

High wear and surface roughness of X-tra fil can also be due to the presence of TEGDMA monomer in its composition, which decreases the viscosity of the resin matrix. TEGDMA is more susceptible to hydrolysis than bis-GMA and bis-EMA; therefore, it increases the water sorption by the matrix, which subsequently increases the wear and surface roughness of composite. Degradation of the resin matrix by hydrolysis depends on the degree of conversion and monomer composition [26]. Although Z250 and EverX-Posterior also contain TEGDMA, the TEGDMA percentage in their composition is relatively less compared to that of X-tra fil composite which might explain the superior results obtained for these composites [31]. Also, the size, volume, distribution, and chemical composition of fillers, and properties of the resin matrix and photo-initiator are among other influential factors on wear [7, 32]. The glass transition temperature is another factor affecting the degree of wear, which is defined as the temperature at which a material is converted from the solid state to elastic state. The glass transition is an important property of the cured matrix of resin composites. Inadequate polymerization and subsequently low degree of conversion of the composite result in a low final glass transition [27]; it is believed inadequate polymerization of the composite results in a lower degree of conversion and produces a polymer which is more susceptible to softening and wear due to less a cross-linked structure [33]. However, a previous study evaluating the degree of conversion of tested bulk-fill composites (Filtek Bulk-Fill, X-tra fil, EverX-Posterior, and SonicFill 2) reported that all composites showed degree of conversion above the standard threshold of 55–65% [34].

The present results revealed that among the tested bulk-fill composite resins, EverX-Posterior and Filtek Bulk-Fill had the lowest wear; the weight loss in these two composite resins was significantly different from that in X-tra fil. Low wear of EverX-Posterior can be attributed to better stress distribution in the resin matrix due to the fibers present in its composition. Also, these fibers can stop crack propagation [35]. Our results, however, were different from those of Kumar et al., [36] who assessed the wear resistance of several bulk-fill composite resins compared with gold. They reported that Tetric N Ceram and EverX-Posterior had greater wear than cast gold. Furthermore, Hamouda et al. [37] evaluated the mechanical properties of nano-filled composite resins and demonstrated that Filtek Supreme nano-filled composite resin had higher wear resistance than a hybrid composite. Lower wear of nano-filled composite resins can be explained by the smaller size of their filler particles (5–20 nm) and their higher filler content.

The current results showed weight loss of specimens following attrition wear, which can be due to the fact that in the process of attrition, direct localized contact of specimens with the cusp tip of the opposing tooth would result in localized micro-fractures at the respective site.

Yesil et al. [38] assessed the wear resistance of a nano-filled composite. The specimens underwent abrasion wear as well as attrition wear caused by contact with a natural tooth. The results revealed that the magnitude of wear due to attrition was higher than that due to abrasion.

## Conclusion

Within the limitations of the present study, bulk-fill composite resins might be a reliable alternative used in posterior areas with no concern regarding their attrition wear or surface roughness because their attrition wear, surface roughness and weight loss were comparable to those of the tested conventional composite resin. However, further in vivo and in vitro studies are required to confirm our results. Moreover, evaluating the samples before and after attrition wear using scanning electronic microscopy would be recommended in future studies.

## Supplementary Information

**Additional file 1:** Supporting data of the present study.

## Data Availability

All data generated or analyzed during this study are included in this published article and Additional file 1.
